# Tramadol and Tapentadol Induce Conditioned Place Preference with a Differential Impact on Rewarding Memory and Incubation of Craving

**DOI:** 10.3390/ph16010086

**Published:** 2023-01-07

**Authors:** Joana Barbosa, Sandra Leal, Frederico C. Pereira, Ricardo Jorge Dinis-Oliveira, Juliana Faria

**Affiliations:** 1 TOXRUN—Toxicology Research Unit, University Institute of Health Sciences—CESPU (IUCS-CESPU), 4585-116 Gandra, PRD, Portugal; 2 UCIBIO-REQUIMTE—Applied Molecular Biosciences Unit-Network of Chemistry and Technology, Laboratory of Toxicology, Department of Biological Sciences, Faculty of Pharmacy, University of Porto, 4050-313 Porto, Portugal; 3 CINTESIS@RISE—Center for Health Technology and Services Research of the Health Research Network, MEDCIDS—Department of Community Medicine, Information and Health Decision Sciences, Faculty of Medicine, University of Porto, Rua Dr. Plácido da Costa, 4200-450 Porto, Portugal; 4 Institute of Pharmacology and Experimental Therapeutics/iCBR—Coimbra Institute for Clinical and Biomedical Research, Faculty of Medicine, University of Coimbra, 3000-354 Coimbra, Portugal; 5 Department of Public Health and Forensic Sciences, and Medical Education, Faculty of Medicine, University of Porto, 4099-002 Porto, Portugal; 6 MTG Research and Development Lab, 4200-604 Porto, Portugal

**Keywords:** tramadol, tapentadol, conditioned place preference, rewarding memory, incubation of craving, abuse, drug misuse, dependence, psychopharmacology

## Abstract

Tramadol and tapentadol, synthetic opioids commonly prescribed for moderate-to-severe pain, have a unique pharmacology that optimizes their analgesia and safety. However, they are not devoid of risks, presenting addictive, abuse, and dependence potential. While tramadol-reinforcing properties have been documented by various studies with human and animal models, including conditioned place preference (CPP) assays, no similar studies have been performed with tapentadol. In the present study, we performed CPP assays by intraperitoneally administering Wistar rats with a tramadol/tapentadol therapeutic dose. Animal permanence and the number of entries in the CPP compartments were recorded in the preconditioning phase and then 1 (T1), 7 (T7), and 14 (T14) days after conditioning. Both opioids induced a change in place preference (T1), suggesting that they have short-term reinforcing properties. However, only tramadol was associated with place preference retention (T7 and T14), with an increase in the number of entries in the opioid-paired compartment (T1 and T7), showing that it causes rewarding memory and incubation of craving. The results indicate that at therapeutic doses: (1) both drugs cause short-term rewarding effects and (2) as opposed to tramadol, tapentadol does not cause CPP retention, despite its higher central nervous system activity and stricter scheduling.

## 1. Introduction

**Figure 1:**
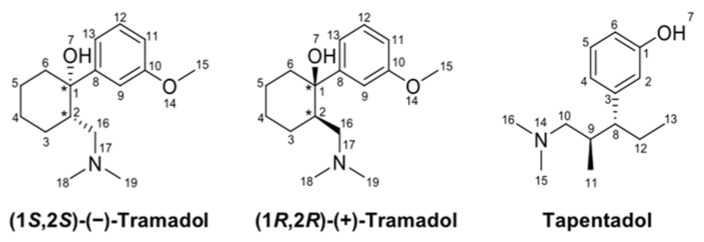
Chemical structures of tramadol and tapentadol. The chemical structures of tramadol (+)- and (−)-enantiomers are depicted. The stereocenters are depicted with asterisks. Atom positions are sequentially numbered.

Tramadol and tapentadol, two of the most commonly prescribed opioids worldwide [1,2], are structurally related synthetic drugs (Figure 1) used in the treatment of moderate to severe, acute, and chronic pain. Besides acting as µ-opioid receptor (MOR) agonists, they display monoaminergic properties, explaining their classification as atypical opioids. Such dual and synergistic mechanisms of action allow them to effectively provide analgesia, while comparatively improving their analgesic and safety profiles [3,4,5]. 

There are, however, some remarkable differences between both drugs. While tramadol, marketed as a racemate, inhibits both serotonin and noradrenaline reuptake, tapentadol acts mainly by inhibiting that of noradrenaline [3,4,5]. Tramadol analgesia is highly dependent on its conversion into *O*-desmethyltramadol (M1), its active metabolite, by the CYP2D6 isoenzyme; its opioid, noradrenergic and serotonergic activities reside mainly in the (+)-M1, (−)-tramadol and (+)-tramadol enantiomers, respectively [6]. Owing to its serotonergic component, and contrarily to tapentadol, it is associated with serotonin syndrome, besides the classic opioid-related adverse events [7]. In turn, a single parent molecule ensures tapentadol opioid and noradrenergic mechanisms of action. Requiring no bioactivation, tapentadol avoids interindividual therapeutic variability due to genetic and metabolic variations [6]. Its noradrenergic component has been associated with anti-apoptotic and pro-neurogenic effects, supporting its use in neuropathic pain states [4,8,9,10,11,12,13,14]. Tapentadol shows higher MOR affinity than parent tramadol, which makes the former more effective in the analgesia of pain requiring the efficacy level of strong opioids and the latter more suited to the treatment of multiple pain types where a strong opioid component is not required or desired [6]. Tapentadol is two to five times more potent than tramadol in distinct animal pain models [6]. 

While the rates of abuse and dependence are considerably lower for tramadol and tapentadol than for other opioid peers, they are not devoid of risk, as substantiated by several reports [3,4,15,16,17,18,19,20,21,22,23]. Although tramadol is listed as a Schedule IV controlled substance in the USA, with the rates of abuse and misuse being lower than those of other prescription opioids, it is emerging as a drug of abuse in the Middle East and African countries, as well as in other developing nations [7]. Users report a sense of happiness but also cognitive alterations [4,24].

Tramadol is actively transported across the blood-brain barrier, whilst tapentadol has no active transport mechanism known [6,25]. This may contribute to explaining both tapentadol’s higher central nervous system (CNS) activity and detrimental effects. Indeed, although less than with other potent opioids, tapentadol’s prominent MOR agonism is associated with mood alterations and euphoria and, thus, with dependence and abuse potential [26,27]. A study with occasional opioid users showed that, unlike tramadol, therapeutic doses of tapentadol led exclusively to positive subject-rated effects, with faster onset and offset than other opioids, which may contribute to a greater frequency of use [28,29]. A recent web-based survey on tapentadol non-medical use revealed that, besides pain relief, it is consumed for multiple psychotropic effects, including relaxation, reduction in depression or anxiety, and getting high; a subgroup of participants even reported hallucinogenic side effects at higher doses [30]. Such potential to induce physical and psychological dependence, supported by rewarding and reinforcing effects comparable to those of morphine, underpinned its classification as a Schedule II substance in the USA [7,28].

Opioids activate the mesolimbic reward system, generating signals in the ventral tegmental area that lead to dopamine release in the nucleus accumbens shell; this, in turn, causes a feeling of pleasure that has been suggested as a biochemical cause of dependence [31]. A number of studies, combining biochemical and behavioral approaches, have experimentally shown that tramadol induces rewarding effects corresponding to psychological dependence [18,31,32,33,34,35,36]. Such effects are likely due but not limited to M1-mediated MOR activation [37,38]. Tapentadol’s higher CNS activity, as well as its stricter scheduling, underlines the pertinence of similar studies with this opioid. Nonetheless, they are paradoxically missing. 

Therefore, in the present study, we aimed to comparatively assess tramadol and tapentadol’s rewarding and reinforcing properties, at a therapeutic dose, by using the conditioned place preference (CPP) assay, a non-clinical animal paradigm of addiction potential [39,40]. The relevance of the results is particularly emphasized by the worldwide increasing rates of prescription and misuse of both drugs.

## 2. Results

### 2.1. Repeated Exposure to Tramadol and Tapentadol Leads to Changes in Place Preference

**Figure 2:**
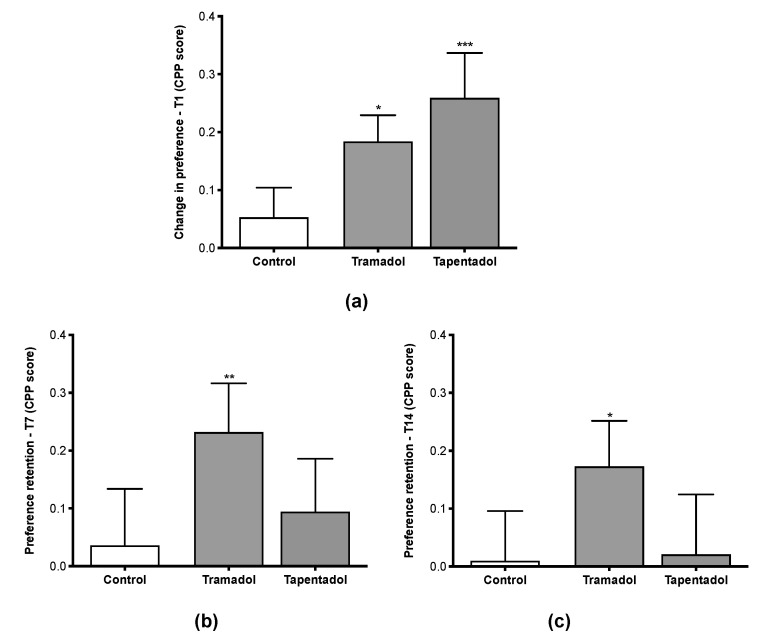
Change in place preference at T1 (**a**), T7 (**b**) and T14 (**c**) time points. Change in preference was assessed by measuring the difference between CPP scores 1 (**a**), 7 (**b**) and 14 (**c**) days after the last drug administration (conditioning phase) and the CPP score in the preconditioning phase. In the conditioning phase, rats received daily intraperitoneal injections of 50 mg/kg tramadol or tapentadol, alternating with normal saline, for 8 days, and were immediately allowed to stay in a pre-determined (drug-paired: non-preferred; saline-paired: preferred) compartment. Control animals received daily intraperitoneal injections of normal saline for the same period. Results were recorded with the PPCWIN software, version 2.0 (Panlab, ref. 76-0011), and are expressed as means ± SD (*n* = 9). * *p* < 0.05; ** *p* < 0.01; *** *p* < 0.001.

CPP assays are used to analyze drug-positive reinforcing effects. As such, we used the CPP paradigm to evaluate the putative induction of psychological dependence or abuse liability by tramadol or tapentadol. In this context, animals received intraperitoneal (i.p.) injections of tramadol or tapentadol, alternating with normal saline, for 8 days; each opioid was administered at a 50 mg/kg dose, corresponding to the maximum recommended daily dose. The results were compared with those from the control group (injected with saline solution). The results obtained one day after the last administration (Figure 2a) suggest that both tramadol (*p* < 0.05) and tapentadol (*p* < 0.001) cause a significant change in place preference, possibly associated with reinforcing properties. The change in place preference is reflected in the increase in the time spent in the initial non-preferred (drug-paired) compartment and derives from the rewarding effects induced by the drugs. After tramadol exposure, the animals spent, on average, 2.8 ± 0.7 additional mins in the non-preferred compartment compared with the period spent in the preconditioning phase (before drug administration). A similar result was observed for animals administered with tapentadol, for which an increase of 3.9 ± 1.2 min was detected in the permanence time in the non-preferred compartment. 

### 2.2. Tramadol, but Not Tapentadol, Causes CPP Memory for at Least 14 Days upon Conditioning

In order to evaluate the long-term rewarding memory in tramadol- and tapentadol-treated animals, they were again put in the CPP apparatus 7 and 14 days after the last conditioning session (T7 and T14, respectively), and the time spent in each compartment was recorded (Figure 2b,c). Animals exposed to tramadol showed a significant maintenance of the place preference score both 7 (*p* < 0.01) and 14 (*p* < 0.05) days after the last drug administration (Figure 2b,c, respectively). Indeed, the time spent in the non-preferred compartment increased on T7 (3.5 ± 1.3 min) and T14 (2.6 ± 1.2 min).

In contrast, no significant differences were detected between animals administered with saline and tapentadol, suggesting that this opioid has lower or even no potential to induce long-term rewarding memory when compared with tramadol.

### 2.3. Tramadol, but Not Tapentadol, Shows Craving Properties

**Figure 3:**
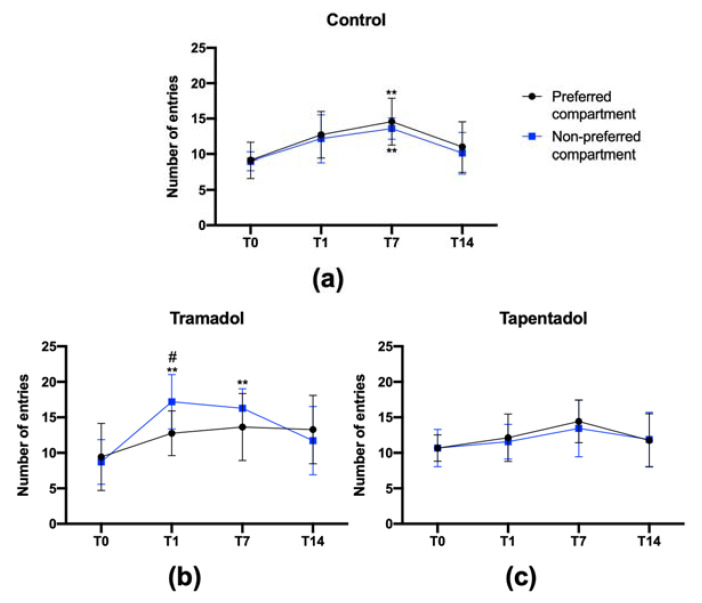
Number of entries in each compartment (preferred (saline-paired) compartment and non-preferred (drug-paired) compartment) at each time point—preconditioning phase (T0), and 1, 7 and 14 days after the last drug administration (T1, T7 and T14, respectively). In the conditioning phase, rats received daily intraperitoneal injections of 50 mg/kg tramadol (**b**) or tapentadol (**c**), alternating with normal saline, for 8 days. Control animals received daily intraperitoneal injections of normal saline for the same period (**a**). Results were recorded with the PPCWIN software, version 2.0 (Panlab, ref. 76-0011), and are expressed as means ± SD (*n* = 9). ** *p* < 0.01 compared with T0; # *p* < 0.05 between the preferred and non-preferred compartment (same time point).

Following the model proposed by Sun and colleagues [40,41], the number of entries in each compartment was quantified to determine whether tramadol or tapentadol showed reinforcing and craving properties. In the control group (Figure 3a), the number of entries in the preferred and non-preferred compartments was not different at each time point—in the preconditioning phase (T0) and 1, 7, and 14 days upon the last drug administration (T1, T7, and T14, respectively). Regarding the T7 time point, a significant increase in the number of entries in both compartments was observed when compared with T0, increasing roughly by 6 entries in the preferred compartment and by 5 entries in the non-preferred compartment (preferred compartment—T0: 9.1 ± 2.5 entries, T7: 14.7 ± 3.6 entries; non-preferred compartment—T0: 9 ± 1.3 entries, T7: 13.6 ± 1.5 entries). 

Animals from the tramadol group (Figure 3b) showed a significant increase in the number of entries in the non-preferred (tramadol-paired) compartment, at the T1 and T7 time points, when compared with T0, increasing approximately by 9 entries on T1, and by 8 entries on T7 (preferred compartment—T0: 7.4 ± 3.9 entries, T1: 12.8 ± 3.2 entries, T7: 13.6 ± 4.7 entries; non-preferred compartment—T0: 8.7 ± 3.1 entries, T1: 17.2 ± 3.8 entries, T7: 16.3 ± 2.7 entries). In addition, the number of entries in the tramadol-paired compartment, at the T1 and T7 time points, was higher when compared with that of the saline-paired compartment. However, on T1, a higher and significant (*p* < 0.05) increase in the number of entries in the non-preferred compartment (roughly 9 additional entries) was observed when compared with that of the preferred compartment (about 5 additional entries), suggesting tramadol had reinforcing properties and that the rats were able to distinguish the tramadol-paired from the saline-paired compartment. Such potential reinforcing properties disappear a few days after the last tramadol administration, given that, on T14, differences in the number of entries are no longer observed between both compartments. 

In contrast, no changes were observed in the tapentadol-treated group (Figure 3c). No significant differences were detected, neither in the number of entries in each compartment, at each time point (T0, T1, T7 and T14), nor in the number of entries in both compartments when compared with T0.

## 3. Discussion

The increase in the use of opioids is a global trend, and the rise in the use, diversion, and misuse of prescription opioids is currently contributing to a worldwide public health concern. Although prescription opioids are considered safe, their potential for abuse, misuse, tolerance, and withdrawal syndrome have not been fully studied yet. Nevertheless, their addiction potential has often been discussed and documented. While tramadol and tapentadol are claimed to have lower abuse liability and dependence potential than their opioid comparators, several data, both from human and in vivo studies, have substantiated their reinforcing properties. However, to the best of our knowledge, there are no similar CPP studies on tapentadol potential for addiction. In this context, the CPP assay, which makes use of animal models and is commonly employed to analyze the rewarding/aversive effect of drugs, including opioids, was used in our study. 

Tramadol is structurally related to codeine and morphine [4,6,42]. In turn, the skeletal structures of morphine, tramadol, and its main metabolite, M1, were used as starting points for tapentadol development [4,13,43], thus validating the comparison between the behavioral effects of these drugs. 

A positive reinforcing effect, associated with opioid receptor activation, was described and confirmed for morphine through the CPP approach [40]. Furthermore, considering their structural and mechanistic similarities with morphine, attention must be focused on the dependence potential of tramadol and tapentadol, which has been underexplored. Since their double and synergistic mechanisms of action encompass opioid agonism, their addiction potential should be considered, even though they have lower MOR affinity than morphine [4,43,44,45].

Different authors have reported that tramadol might have the potential to cause physical and psychological dependence in animals, following the administration of different doses through various routes [32,35,36,46], despite some studies suggesting that it does not induce tolerance or physical dependence, in contrast with morphine [47]. For instance, mice exposed to 80 mg/kg of tramadol (subcutaneous (sc) administration), an analgesic effective dose, as shown by hot-plate and tail-flick analgesia tests, led to a slight tendency towards place preference. Nevertheless, no significant rewarding effects were observed in the CPP assay, suggesting that tramadol has no rewarding properties [48]. However, in this study, the conditioning phase lasted for 4 days, which is half the duration of our analogous phase, corresponding to 2 tramadol administrations only [48]. 

In contrast, through CPP assays, Cha and colleagues have verified that mice treated with tramadol (1.47 and 2.94 mg/kg, i.p.; conditioning phase—8 days) showed clear changes in place preference, and the results were similar to those obtained after methamphetamine administration (1 mg/kg, i.p.) [32]. In addition, similar to our results, Zhang and co-workers reported that tramadol produced significant CPP in adult male Sprague-Dawley rats, at 6, 18 and 54 mg/kg i.p. doses [36]. In turn, Rutten and colleagues also reported similar rewarding effects in the same model, at 1, 3, and 10 mg/kg doses delivered through i.p. injections [46]. They have additionally reported that, in animals under painful conditions, tramadol shows 10 times less potency to induce CPP than in animals under normal conditions [46]. Likewise, Şorodoc et al. [35] observed an induced place preference in male Wistar rats conditioned with tramadol (40 mg/kg i.p.) for 8 days. Another study using Wistar rats showed that 2 and 4 mg/kg i.p. tramadol doses cause a significant CPP effect in a dose-dependent manner [38]. On the other hand, higher tramadol doses (37.5 and 75 mg/kg), administered through i.p. injection, cause a CPP comparable with the effect of morphine (5 mg/kg, sc), in Sprague-Dawley rats [18]. Another study, using adult male balb/C mice administered with 70 mg/kg (sc) tramadol, also reported a significant increase in the time spent in the tramadol-paired compartment [31].

Interestingly, there is evidence suggesting that the combination of tramadol at lower doses (2 mg/kg i.p., a dose without CPP effects) with morphine (0.125, 0.25, 0.5 mg/kg, sc) or buprenorphine (0.01, 0.0316, 0.1 mg/kg, sc) increased the retention time of both drugs, compared with those of morphine or buprenorphine alone, potentiating their rewarding effects in an additive manner [36]. For this reason, the simultaneous use of multiple opioids may increase the dependence risk [36]. Additionally, tramadol physical dependence was also suggested through jumping tests in animals treated with morphine upon tramadol pre-treatment [32].

Concerning the retention of the CPP effect, some authors did not detect a CPP retention 14 days after the last injection of tramadol in the conditioning phase [36]. Nevertheless, our results showed that, in our in vivo model, the memory of the tramadol-induced CPP effect lasted at least 14 days after the last administration. In contrast, tapentadol did not lead to CPP retention at the dose tested (Figure 2b,c). Several studies suggest that tapentadol abuse is infrequent, which may be due to its lower MOR affinity compared with that of classical opioids [49,50]. 

Considering the structural and mechanistic similarities between morphine, tramadol, and tapentadol, as well as the model proposed by Sun and co-workers for the former opioid [40,41], the number of entries in both compartments, at several time points after the last drug administration, was used to analyze the reward memory and the incubation of craving induced by the latter two opioids. CPP assays were established to measure the long-term memory and incubation of craving during abstinence periods, using morphine as a model opioid drug [40,41]. In our study, animals administered with saline solution only (control group) showed a significant increase in the number of entries in both compartments 7 days after the last saline administration, which may be associated with a more intense animal activity on this experimental day, with no place preference. Concerning opioid-treated animals, rats exposed to morphine (10 mg/mL, i.p.) showed an increase in the number of entries in the morphine-paired compartment at least 18 days after the last morphine administration, showing that it causes reward memory at least for this period [40]. In our assays, following the same experimental approach, the results evidenced that tramadol causes reward memory for 7 days, but it declines over time, with no differences being detected on day 14. Given that, on T1 and T7, the animals were not administered with tramadol, they may have learned that the tramadol-paired compartment no longer had a rewarding effect, similar to what has been previously suggested by experiments with morphine [41]. Furthermore, our results suggest that tramadol causes longer drug memory, since place preference was preserved for 14 days. In addition, the increase in the number of entries in the tramadol-paired compartment on T1 and T7 suggests a motivational state associated with a drug craving, as observed in animals treated with classical opioids [40]. 

**Figure 4:**
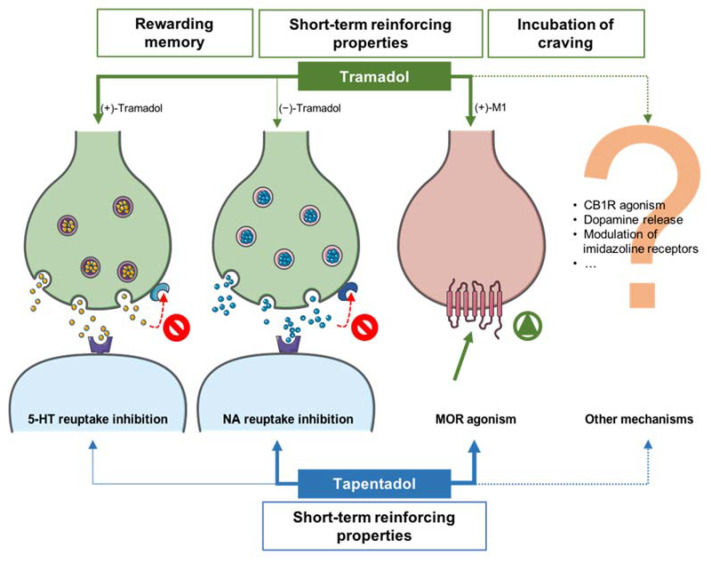
Summary of tramadol and tapentadol mechanisms of action and CPP behavioral effects. Arrow thickness depicts the approximate relative intensity of each effect. Regarding tramadol, the parent drug or metabolite enantiomer predominantly responsible for each effect is specified. 5-HT: 5-hydroxytryptamine/serotonin; CB1R: cannabinoid receptor 1; M1: *O*-desmethyltramadol; MOR: µ-opioid receptors; NA: noradrenaline. Red circumferences with diagonal lines: 5-HT/NA reuptake inhibition; green circumference with triangle: MOR activation.

Conversely, in the tapentadol group, a higher change in place preference, when compared with the control group, was detected in T1 (Figure 2a), possibly due to its higher CNS activity. Yet, despite such properties and the comparatively more pronounced effects of tapentadol on brain structures [20,23], no alterations were observed in the number of entries in both compartments throughout the study. Tapentadol’s mechanism of action, as well as the lack of changes in the brain circuitry mediating reward and motivation, might be associated with its lower propensity to induce addiction [39,49,50,51]. Its relatively low MOR affinity has been claimed as the main reason behind its lower abuse potential [4,39,52]. In this sense, the fact that tramadol and the (+)-M1 enantiomer present lower and higher MOR affinities than tapentadol, respectively, support the idea, advocated by other authors, that M1-mediated MOR activation is a key player on tramadol-induced psychological dependence [37,38] (Figure 4).

Altogether, the results suggest that tramadol appears to have a higher potential to induce long-term memory and incubation of craving than tapentadol. In fact, the risk of tramadol dependence, particularly in subjects with an abuse history, was underlined by different authors [4,53]. Prolonged tramadol use, particularly in pain-free conditions, may increase the risk of dependence [46].

Lastly, it is also important to emphasize that, although tramadol-induced conditioning could be associated with MOR agonism, other mechanisms, such as the agonism of the cannabinoid receptor 1 (CB1R), located in the nucleus accumbens (NAc), were already implied in tramadol reinforcing effect [38] (Figure 4). Moreover, changes in dopamine levels in the NAc were also reported in animals exposed to 75 mg/kg (i.p.) and 70 mg/kg (sc) tramadol [18,31]. Since tramadol inhibits serotonin reuptake and promotes dopamine release, and considering the involvement of these neurotransmitters in mood alterations, the contribution of these effects to addiction should not be disregarded [54] (Figure 4). The increase in dopamine levels, caused by tramadol, can be blocked through nalbuphine co-administration, which leads to CPP extinction [31]. Hence, nalbuphine administration could represent a strategy to prevent tramadol’s psychological dependence [31]. A more recent study, using simultaneous exposure to tramadol and imidazoline receptor ligands, suggested a potential role of such receptors in tramadol-induced CPP [35].

In order to gain further insights into tramadol and tapentadol dose-effect relationships, including eventual dose dependence, additional opioid doses—particularly higher doses—should be assayed through the CPP approach, which remains a future prospect of the present study. Likewise, combined drug exposure assays (for instance, with other monoamine reuptake and MOR modulators) will provide additional mechanistic data on tramadol/tapentadol rewarding properties, CPP memory, and putative further aspects influencing their magnitude.

## 4. Materials and Methods

### 4.1. Chemicals

Tramadol hydrochloride was purchased from Sigma-Aldrich (St. Louis, MO, USA), while tapentadol hydrochloride was obtained from Deltaclon (Madrid, Spain). Sodium thiopental was provided by B. Braun Medical (Queluz de Baixo, Oeiras, Portugal). Normal saline (0.9 g/L (*w/v*) NaCl) was used as a vehicle for the administration.

### 4.2. Experimental Models, Animal Housing and Handling

27 male Wistar rats, aged 8 weeks and weighing approximately 250 g, were supplied by the i3S animal facility (Porto, Portugal). 

Animals were housed in acrylic cages, two per cage, in an environment enriched with wood chips, paper towels, and tubes and kept under standard controlled conditions (22 ± 2 °C, 50–60% humidity, 12/12 h light/dark cycles) throughout the investigation. They were allowed unlimited access to tap water and rat chow (standard short and middle-period maintenance formula for rodents, reference 4RF21, Mucedola/Ultragene (Milan, Italy)). A quarantine period of at least one week was observed before experimental assays. 

Once all the assays were finished, animals were sacrificed through i.p. injection of 60 mg/kg sodium thiopental, dissolved in a saline solution.

Animal experimentation followed the European Council Directive (2010/63/EU) guidelines, transposed into Portuguese law (Decree-Law no. 113/7 August 2013). The experimentation has also received approval from the Ethics Committee of CESPU, Gabinete para a Investigação e a Inovação (GI2), Gandra, PRD, Portugal (processes no. PI-2RL 2021 and GI2-CESPU 2022), and complied with the National Ethics Council for the Life Sciences (CNECV) guidelines. 

### 4.3. Conditioned Place Preference Assays

Rats were randomly assigned to 3 groups of 9 animals each. The sample size was determined using the G*Power software, version 3.1.9.6 (Heinrich-Heine-Universität Düsseldorf, Düsseldorf, Germany), assuming a significance level of 0.05, an 80% power, and effect size values adjusted accordingly with results reported in the literature for similar behavioral tests. 

#### 4.3.1. Apparatus and Environmental Conditions

CPP assays were conducted in an automated place reference box—black and white, reference LE892 (76-0218), provided by Panlab (Barcelona, Spain). The apparatus was composed of a left white compartment and a right black compartment, both of the same sizes, interconnected by a central, smaller gray corridor. The three compartments were separated by manually controlled guillotine doors. The front panel of the black and white compartments were smoked translucent, whilst that of the gray compartment was opaque. To create equally inviting environments and to facilitate their distinction, a rough floor was placed in the white compartment, while a smooth one was placed in the black compartment; the gray corridor also had a smooth floor. Dimensions were as follows: black/white compartment (each)—300 × 300 × 340 mm, *w* × *d* × *h*; corridor: 80 × 100 × 340 mm, *w* × *d* × *h*; doors—100 × 140 mm, *w* × *h*. The animal position was detected using a weight transducer technology that was incorporated into the apparatus. The time spent and the transitions/number of entries in each compartment were recorded for each animal, using the PPCWIN Software Associated to Automated Boxes (up to 8 units), version 2.0, reference 76-0011 (Panlab). Before and between sessions, the apparatus was carefully disinfected and deodorized with 70% (*v*/*v*) ethanol, which was then allowed to dry completely. CPP assays were performed under uniform lighting and with no sources of visual, auditive, or olfactive disturbance.

#### 4.3.2. Preconditioning

On each of 3 consecutive days (days 1–3), rats were put in the gray corridor and given 15 min to freely explore the apparatus, enabling the assessment of unconditioned preference. On day 3, the time spent on each compartment was recorded and used as a baseline (T0).

#### 4.3.3. Conditioning

During 8 consecutive days (days 4–11), rats received daily i.p. injections of 50 mg/kg tramadol or tapentadol, delivered in 1 mL-units and immediately placed in the respective non-preferred compartment (as assessed in the preconditioning phase) for 40 min. Doses are equivalent to tramadol and tapentadol maximum daily doses for humans, as previously described [20,21,22,23,55,56,57]. Animals were weighed every 3–4 days, regarding dose adjustment to total body weight. Assays were designed so that animals with opposite non-preference were paired and simultaneously conditioned; in each pair, each rat was put in the respective non-preferred compartment, paired with drug treatment. Access to the other compartments was blocked by inserting the doors into the respective guides. Animals from the same pair were switched every day, so that the drug was injected every other day, alternating with days where they were administered with i.p. injections of 1 mL vehicle (saline solution) only. In turn, rats from the control group were injected daily with 1 mL saline solution during the entire conditioning phase. Drug/saline solution administration took place at the same time each day.

#### 4.3.4. Postconditioning

On day 12, in the absence of drug or saline administration, rats were placed in the gray corridor and allowed free access to all compartments for 15 min. The time spent in each compartment was recorded and used as a test line (T1). The number of entries in each compartment was also registered. The CPP score was calculated as the fraction of time spent in the drug-paired (non-preferred) compartment vs. the total time spent in both conditioning compartments.

Similar postconditioning tests were additionally performed on days 19 and 26 (7 and 14 days upon the last drug administration—T7 and T14, respectively).

### 4.4. Statistical Analysis

Statistical analysis was performed through an Analysis of Variance (ANOVA), followed by Dunnett’s multiple comparisons test as a post-hoc analysis. In all determinations, results were compared with those from the control animals and injected with a saline solution. Comparative analysis of data within the same time points was performed as unpaired, two-tailed Student *t*-tests. Data are presented as means ± SD and probability values of *p* < 0.05 were considered statistically significant. Graphic plotting and all statistical tests were performed with GraphPad Prism^®^, version 8.3.1 (GraphPad Software, LLC, San Diego, CA, USA).

## 5. Conclusions

The present study demonstrated that tramadol and tapentadol induce CPP, with tapentadol causing a more pronounced change in place preference when compared with the control group. Nevertheless, only tramadol showed the potential to induce drug memory and incubation of craving several days after the last drug administration. Collectively, the results suggest that tapentadol has a lower propensity to induce dependence than tramadol.

These data alert the scientific and medical communities to the potential tramadol and tapentadol dependence and underline the recommendation for their careful prescription.

## Data Availability

Data are contained within the article.
